# Age-Related Excessive Tortuosity of the Right Brachiocephalic Artery in Patients Being Evaluated for Transcatheter Aortic Valve Replacement (TAVR): Potential to Affect Alternate Access

**DOI:** 10.7759/cureus.115579

**Published:** 2026-09-01

**Authors:** Kyrillos Girgis, Prashant K Gupta, Danielle Retcho, Divine Dele, Aarti Gupta, Rajiv Tayal, Nishant Sethi, Arash Salemi, Sergio Waxman, Najam Wasty

**Affiliations:** 1 Cardiology, Newark Beth Israel Medical Center, Newark, USA; 2 Radiology, Newark Beth Israel Medical Center, Newark, USA; 3 Internal Medicine, Newark Beth Israel Medical Center, Newark, USA; 4 Cardiology, The Valley Hospital, Paramus, USA; 5 Cardiology, Commonwealth Health Physician Network, Scranton, USA; 6 Cardiothoracic Surgery, Robert Wood Johnson Barnabas (RWJBarnabas) Health, West Orange, USA

**Keywords:** alternate access for tavr, brachiocephalic artery, tavr, tortuosity, vascular access

## Abstract

Background

We hypothesized age-related excessive tortuosity of the right brachiocephalic artery (RBCA) in patients undergoing transcatheter aortic valve replacement (TAVR) evaluation.

Methods

This single-center, retrospective, cross-sectional study evaluated 100 consecutive patients aged ≥70 undergoing TAVR workup. Contrast-enhanced computed tomography (CT) angiography of the chest, abdomen, and pelvis was performed, and three-dimensional volume-rendered reconstructions were generated. Tortuosity of the RBCA was quantified based on the number and degree of angulations (≥45° or ≥90°).

Results

Of the 100 patients, the mean age was 80.6 years, there were 53 females, 37 diabetics, and 99 hypertensives, the mean low-density lipoprotein (LDL) was 81.8 mg/dL, the mean ejection fraction was 57.1%, and the mean of the mean aortic valve gradients was 33.6 mmHg. About 88% of the patients had a varying combination of at least up to two angulations ≥45° and/or ≥90°, while 47% had at least two angulations ≥90°.

Conclusions

Incidence of excessive tortuosity of the RBCA in the elderly being evaluated for TAVR is indeed very high; 88% of the patients had a varying combination of at least up to two angulations ≥45° and/or ≥90°, while 47% had at least two angulations ≥90° (normal in young healthy subjects being no angulations at all ≥45° and/or ≥90°).

This excessive RBCA tortuosity may affect the ease and success of procedures performed using the right axillary approach, particularly for TAVR alternate access (percutaneous axillary (PercAx)).

Larger studies are needed to corroborate our findings not only in patients with aortic stenosis being evaluated for TAVR but also in this age group in general, since such excessive RBCA tortuosity may, to some degree, explain the increased incidence of radial-to-femoral conversion with advancing age.

## Introduction

Severe aortic stenosis is one of the most common valvular diseases affecting older adults, and transcatheter aortic valve replacement (TAVR) has transformed its management, extending effective treatment across the full spectrum of surgical risk [1]. As TAVR volumes continue to grow, an increasing number of very elderly and anatomically complex patients are being evaluated for the procedure, making the careful characterization of potential vascular access routes an increasingly important component of pre-procedural planning.

The prevalence and severity of right brachiocephalic artery (RBCA) tortuosity in elderly patients undergoing TAVR evaluation remain poorly characterized; we hypothesized there may exist an age-related excessive tortuosity of the RBCA in patients undergoing TAVR evaluation.

TAVR has become a standard therapeutic option for patients with severe aortic stenosis not only in the elderly and those at high surgical risk as in the past [1]. While transfemoral access is the most common approach, anatomical challenges sometimes may necessitate alternate access strategies, including percutaneous axillary (PercAx) access [2].

Selection of the optimal access route for TAVR relies on a systematic, heart team-based review of the iliofemoral, subclavian/axillary, carotid, and other candidate access vessels on pre-procedural computed tomography (CT) angiography, with current expert consensus recommending an individualized approach whenever the transfemoral route is anatomically unsuitable [3]. Among the available alternate access options, PercAx has become an increasingly favored strategy given its extrathoracic location, relative resistance to atherosclerotic calcification compared with the iliofemoral vessels, and outcomes that can approach those of transfemoral access in appropriately selected patients [2,3].

The RBCA comprising the brachiocephalic artery and its continuations, the right subclavian and axillary arteries, is a common alternate access for TAVR. Age-related structural changes, including vessel elongation, tortuosity, and calcification, may complicate device navigation and delivery and lead to procedure risk [4].

With advancing age, the arterial wall undergoes progressive structural remodeling characterized by fragmentation of elastin fibers, accumulation and cross-linking of collagen, and loss of vascular elasticity; these degenerative changes, compounded by chronic hemodynamic stress and hypertension, promote elongation and tortuosity of previously straight arterial segments [5]. Quantitative imaging studies of the aorta and of the carotid and vertebral circulations have similarly demonstrated a consistent, positive correlation between advancing age and vessel tortuosity [6], supporting the broader concept that the RBCA may be subject to the same age-related structural remodeling recognized in other arterial beds.

Prior studies have evaluated tortuosity in the aorta, iliac, and femoral arteries [7,8]. However, systematic data on RBCA tortuosity are scarce. Understanding the patterns and degree of tortuosity in this arterial segment may be helpful in PercAx TAVR planning.

Beyond TAVR, excessive tortuosity of the subclavian and innominate segments is also a well-recognized technical obstacle during transradial coronary and peripheral interventions, where it can contribute to procedural failure, prolonged fluoroscopy and contrast exposure, and, rarely, vascular injury [9]. Given the anatomic continuity of the RBCA with the subclavian and axillary arteries, characterizing its tortuosity in the same elderly population being evaluated for TAVR may have implications that extend beyond alternate access planning to transradial procedures more broadly.

This article was previously presented as a poster abstract at the Society for Cardiovascular Angiography and Interventions (SCAI) 2026 Scientific Sessions in Montreal, Canada, on April 24, 2026.

## Materials and methods

Study design and population

This single-center, retrospective, cross-sectional study included patients aged ≥70 years undergoing TAVR evaluation at Newark Beth Israel Medical Center in Newark, New Jersey, between January 2024 and June 2025. Patients with any prior cardiothoracic or aortic interventions were excluded. Ethical approval was obtained from the Newark Beth Israel Medical Center Institutional Review Board (approval number: 2026.06) as per institutional policy, which waived the requirement for informed consent given the retrospective nature of the study.

Imaging protocol

All patients underwent contrast-enhanced CT angiography of the chest, abdomen, and pelvis following the standard TAVR protocol. Chest CT angiography was acquired in axial, coronal, and sagittal planes using the standard protocol.

3D volume rendering (VR) and vessel reconstruction

High-quality 3D VR images were later generated using the Aquarius iNtuition software (TeraRecon, Inc., Durham, NC, USA) by loading the relevant DICOM dataset into the application, typically thin-section (0.65 mm) axial images of contrast-enhanced CT angiography of the chest for vascular reconstructions.

Upon loading, the dataset is reformatted using the curved planar reformation (CPR) tool to trace the vessel of interest, which in our study was the RBCA. The centerline extraction was performed using the CPR tool, wherein the vessel of interest was manually traced to generate an accurate centerline, enabling cross-sectional and longitudinal evaluation. This step was critical for optimizing spatial orientation and enhancing the accuracy of subsequent VR.

After CPR alignment, the 3D VR module is activated, allowing for the real-time manipulation of rendering parameters such as opacity, window width/level, and color mapping to highlight vascular contrast. Segmentation tools were applied to isolate regions of interest, while cut planes and rotation controls enabled optimal visualization from multiple perspectives. Final images were then refined using preset or customized rendering protocols before final analysis (Figure 1, Figure 2).

**Figure 1:**
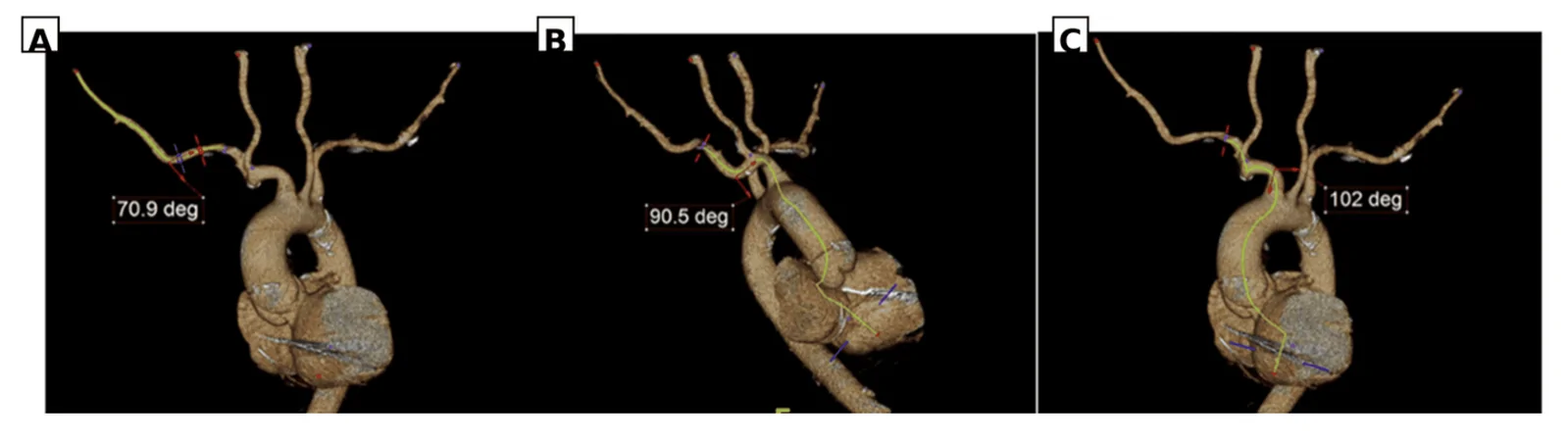
Volume-rendered CT images demonstrating RBCA angulation Volume-rendered CT images in multiple projections from a single patient demonstrating three sequential angulations along the course of the RBCA: (A) 70.9°, (B) 90.5°, and (C) 102°. CT: computed tomography; RBCA: right brachiocephalic artery

**Figure 2:**
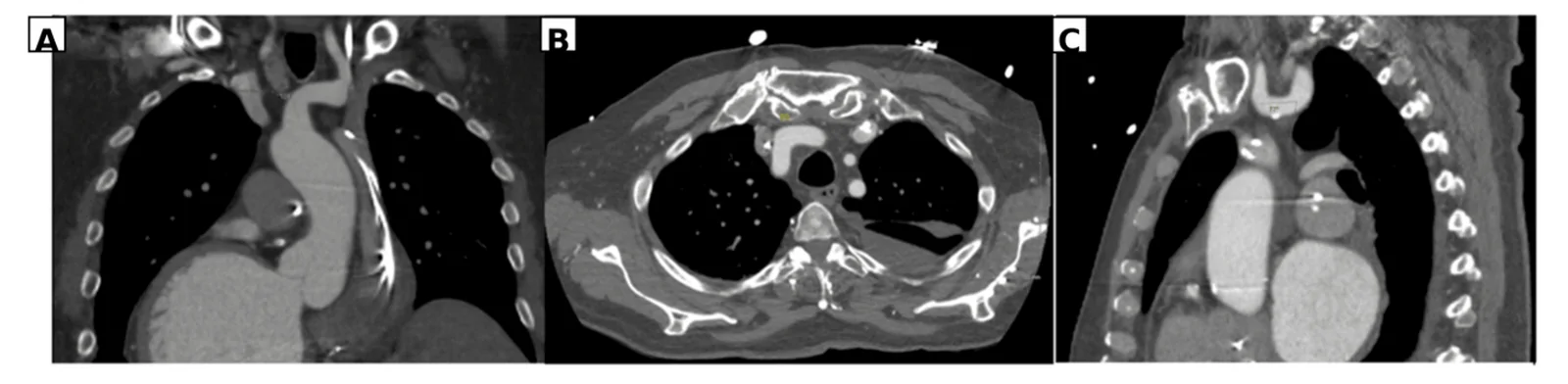
Multiplanar contrast-enhanced CT angiography demonstrating RBCA tortuosity Contrast-enhanced CT angiography images from a different patient than Figure 1 demonstrating excessive tortuosity of the RBCA on multiplanar imaging: (A) coronal, (B) axial, and (C) sagittal. CT: computed tomography; RBCA: right brachiocephalic artery

Tortuosity assessment

The degree of arterial tortuosity was measured using the angle-measuring tool applied to both standard multiplanar (axial, coronal, sagittal) and 3D VR images to ensure accuracy. Tortuosity angles were categorized into two groups: ≥45° (moderate tortuosity) and ≥90° (severe tortuosity).

At each point of curvature identified along the traced RBCA centerline, the angle was defined as the angle formed between straight-line fits to the centerline immediately proximal and distal to that point, that is, the deviation of the vessel's course at each bend, rather than the angle between two specific named branch vessels (e.g., the brachiocephalic and subclavian arteries). This definition was applied consistently on both the 3D VR reconstructions and the corresponding multiplanar images to cross-validate each measurement (Figure 3).

**Figure 3:**
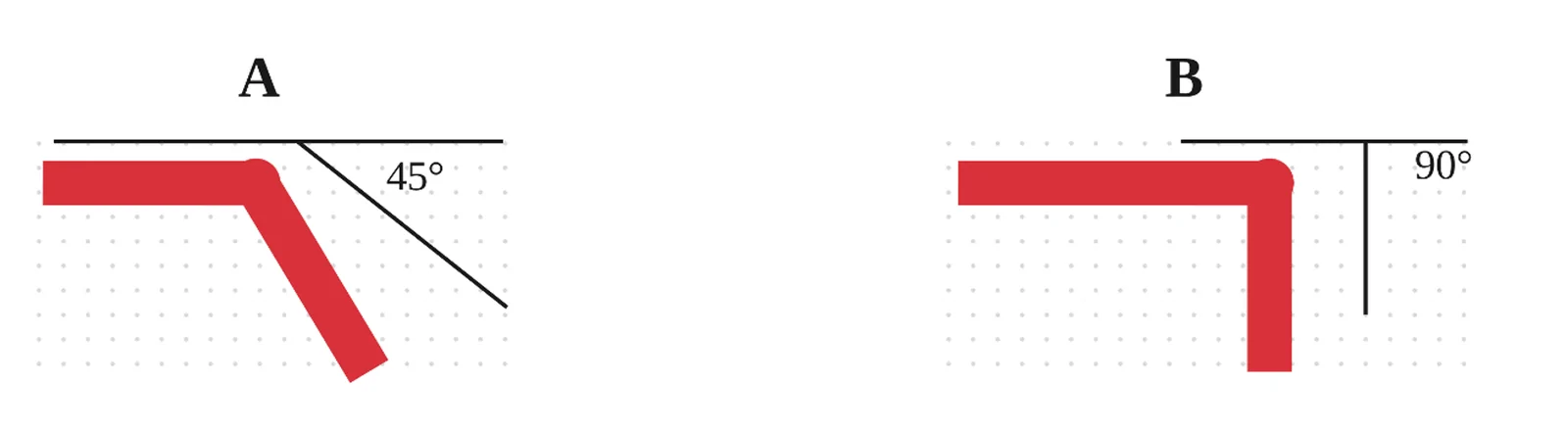
Vessel angle estimation method (A) ≥45° (moderate tortuosity). B ≥90° (severe tortuosity). Schematic depicting the vessel (red) angle estimation method. The black line represents the reference line used to measure the angle between the proximal and distal vessel segments at each point of curvature.

No previously validated tortuosity index exists for the RBCA specifically. The ≥45°/≥90° angle-threshold scheme used here is therefore a pragmatic, study-specific classification rather than an established, externally validated metric (e.g., a tortuosity index or sum-of-angles metric). Reproducing our findings would require applying this same threshold-based scheme, since no other standardized alternative for this arterial segment currently exists.

Statistical analysis

Descriptive statistics were used to summarize all baseline demographic, clinical, and imaging characteristics. Continuous variables were expressed as mean ± standard deviation (SD), and categorical variables were expressed as frequencies and percentages (%). The prevalence of RBCA angulations ≥45° and ≥90° was calculated as a percentage of the overall cohort (N = 100). As this was a descriptive, single-arm cohort study without pre-specified comparison groups, no formal hypothesis testing or inferential statistical analysis was performed, and no dedicated statistical software was used; all summary calculations were performed manually.

## Results

Patient characteristics

The study cohort comprised 100 patients undergoing TAVR evaluation, with a mean age of 80.6 years; 53 patients (53%) were female. Comorbidities included diabetes mellitus in 37 patients (37%), hypertension in 99 patients (99%), and obesity in 35 patients (35%). Thirteen patients (13%) were active smokers. Laboratory evaluation revealed a mean low-density lipoprotein (LDL) cholesterol level of 81.8 mg/dL. Echocardiography showed a mean left ventricular ejection fraction of 57.1% and a mean of the mean aortic valve gradient of 33.6 mmHg (Table 1).

**Table 1:** Baseline characteristics of the study cohort (N = 100) LDL: low-density lipoprotein

Characteristic	Value
Mean age (years)	80.6
Female sex (no. (%))	53 (53)
Diabetes mellitus (no. (%))	37 (37)
Hypertension (no. (%))	99 (99)
Obesity (no. (%))	35 (35)
Active smokers (no. (%))	13 (13)
Mean LDL cholesterol (mg/dL)	81.8
Mean left ventricular ejection fraction (%)	57.1
Mean of the mean aortic valve gradient (mmHg)	33.6

RBCA tortuosity

CT angiography demonstrated that 88% of patients had at least two angulations ≥45° and/or ≥90°, while 47% had at least two angulations ≥90°. The full frequency distribution of the number of angulations meeting each threshold is presented in Table 2. Representative examples of RBCA tortuosity are presented in Figure 1 and Figure 2.

**Table 2:** Frequency distribution of RBCA angulations by threshold (N = 100) RBCA: right brachiocephalic artery

Number of angulations	≥45° (moderate), no. (%)	≥90° (severe), no. (%)
0	12 (12)	19 (19)
1	47 (47)	34 (34)
2	35 (35)	39 (39)
≥3	6 (6)	8 (8)

Figure 1 shows VR CT images (panels A-C) acquired from a single patient, demonstrating three sequential angulations measuring 70.9°, 90.5°, and 102°, respectively, along the course of the RBCA. Figure 2 shows coronal (panel A), axial (panel B), and sagittal (panel C) contrast-enhanced CT angiography images from a different patient, demonstrating excessive tortuosity of the RBCA on multiplanar imaging.

## Discussion

Our analysis demonstrates that excessive tortuosity of the RBCA is highly prevalent among elderly patients undergoing TAVR evaluation. In our cohort, 88% of patients exhibited at least two angulations ≥45° and/or ≥90°, and 47% had at least two angulations ≥90°, indicating that excessive RBCA tortuosity is common in this elderly population. A study by Cha et al. reported that severe tortuosity of the right subclavian artery occurs in approximately 10% of patients undergoing transradial coronary procedures, with older age, hypertension, and female sex identified as significant predictors [4]; our findings additionally demonstrate the continuation of this tortuosity proximal to the subclavian artery into the RBCA which may theoretically lend additional impediment to the ease and success of radial procedures in this age group in general.

The high prevalence of RBCA tortuosity observed in our cohort is consistent with the broader pathophysiology of vascular aging, in which elastin fragmentation, collagen remodeling, and chronic hemodynamic stress promote progressive elongation and angulation of the arterial tree [5]. Prior quantitative imaging studies of the carotid and vertebral circulations have similarly demonstrated a strong positive correlation between advancing age and vessel tortuosity [6], supporting the concept that the RBCA is subject to the same age-related structural remodeling that affects other arterial beds.

Pre-procedural contrast-enhanced CT angiography enables the detailed assessment of vessel diameter, tortuosity, and calcification and currently is the standard practice for transfemoral TAVR [8]. Tortuous or heavily calcified vessels have been associated with a higher risk of vascular complications [7], and CT-derived measures of tortuosity have been shown to independently predict both major and minor access-site complications [8]. Similarly, excessive vessel tortuosity may also influence multiple aspects of PercAx TAVR including safe access, device navigation, and delivery [2,7,8]. Though prior studies demonstrated that tortuosity in the femoral artery, iliac artery, and aorta can impact TAVR procedural success [7,8], RBCA tortuosity has not been systematically evaluated in the context of PercAx TAVR. Our study fills this hiatus by quantifying RBCA tortuosity in an elderly TAVR population, underscoring the importance of detailed pre-procedural imaging and careful planning when PercAx TAVR is being considered.

From a procedural standpoint, the axillary and subclavian arteries are relatively resistant to atherosclerotic calcification compared with the iliofemoral vessels, which has made PercAx an attractive default alternate access strategy when transfemoral access is unsuitable [3,10]. However, excessive tortuosity, independent of calcification, can still meaningfully complicate sheath insertion, guidewire and delivery-system tracking, and overall device maneuverability, potentially increasing fluoroscopy time, contrast use, and the risk of vascular injury [9,11]. Recognition of severe RBCA angulation on pre-procedural imaging may therefore prompt earlier consideration of adjunctive techniques, such as buddy-wire support, stiffer guidewires, or alternative sheath trajectories, or may factor into the heart team's choice between right and left axillary access when both are anatomically feasible.

Our findings may also have relevance beyond TAVR. Older age has repeatedly been identified as an independent predictor of radial-to-femoral access crossover during transradial coronary procedures, with tortuosity of the subclavian and innominate segments cited as a leading technical cause of radial failure [9,12]. By demonstrating that tortuosity frequently extends proximally into the RBCA itself, our findings provide a possible anatomic explanation for the increased crossover rates observed in elderly patients undergoing transradial catheterization, independent of the aortic stenosis or TAVR-specific population studied here.

Limitations of the study

This study has several limitations. First, this was a single-center, retrospective, cross-sectional study with a relatively modest sample size of 100 patients, which may limit the generalizability of our findings to broader, more diverse TAVR populations. Second, RBCA tortuosity was assessed using a single 3D reconstruction software platform (Aquarius iNtuition), and angle measurements, though performed using a validated angle-measuring tool, were not adjudicated by multiple independent readers; inter-observer and intra-observer reliability were therefore not formally assessed. Third, our comparison to a tortuosity-free pattern in young, healthy adults was based on prior published literature rather than a matched internal control group, which may introduce comparability bias. Fourth, this study was cross-sectional in design and did not correlate the degree of RBCA tortuosity with actual procedural outcomes, technical success, or complication rates in patients who subsequently underwent PercAx TAVR or transradial procedures; such outcome data would be necessary to establish the clinical significance of our anatomic findings. Finally, patients with prior cardiothoracic or aortic interventions were excluded, which may have introduced selection bias and does not allow the extrapolation of our findings to patients with prior vascular intervention or surgery.

Additionally, this study examined a single age band (≥70 years) without a younger comparator cohort; as such, our findings characterize the prevalence of RBCA tortuosity within this elderly population but do not, by themselves, statistically demonstrate an age-related trend and should be interpreted as hypothesis-generating rather than confirmatory of an age-tortuosity relationship. Likewise, the anticipated clinical implications of RBCA tortuosity, including its potential effect on PercAx procedural feasibility, fluoroscopy time, and radial-to-femoral access conversion, are drawn by analogy with other arterial beds and were not directly tested against peri-procedural outcomes in this cohort; these associations remain hypothesis-generating pending prospective, outcome-linked studies. Finally, the ≥45°/≥90° angle-threshold classification used to define moderate and severe tortuosity is a pragmatic, study-specific scheme rather than a previously validated tortuosity index, which may limit direct comparability with studies using other tortuosity metrics.

## Conclusions

Prevalence of excessive tortuosity of the RBCA in the elderly being evaluated for TAVR is indeed very high; 88% of the patients had a varying combination of at least up to two angulations ≥45° and/or ≥90°, while 47% had at least two angulations ≥90° (normal in young healthy subjects being no angulations at all ≥45° and/or ≥90°).

Recognition of RBCA excessive tortuosity may be helpful while planning right PercAx TAVR when the standard transfemoral approach is not an option. Review of the already available pre-procedural imaging with contrast-enhanced CT angiography if subjected to three-dimensional reconstruction can aid in identifying challenging anatomy and guiding alternate access strategies.

Larger studies are needed to corroborate our findings not only in patients with aortic stenosis being evaluated for TAVR but also in this age group in general, since such excessive RBCA tortuosity may, to some degree, explain the increased incidence of radial-to-femoral conversion with advancing age.
